# Predicting Risky Behavior Among Pregnant Women in Eastern African countries using Pooled Demographic and Health Survey (DHS) Data: An Explainable Machine Learning Approach

**DOI:** 10.1186/s12884-026-09725-7

**Published:** 2026-08-10

**Authors:** Halid Worku Jemil, Sonia Worku Semayneh, Altaseb Beyene Kassaw, Anmut Endalkachew Bezie

**Affiliations:** 1 Department of Health Informatics, College of Medicine and Health Science, Wollo University, P.O. Box 1145, Dessie, Ethiopia; 2 Department of Oncology, Addis Ababa University College of Health Science, Tikur Anbessa specialized hospital, Addis Ababa University, Addis Ababa, Ethiopia; 3 Department of Biomedical Science, College of Medicine and Health Science, Wollo University, Dessie, Ethiopia; 4 Department of Occupational and Safety, College of Medicine and Health Science, Wollo University, Dessie, Ethiopia

**Keywords:** Machine Learning, Risky Behavior, Light Gradient Boosting Machine (LGBM), Pregnant Women, Shapley Additive Explanation (SHAP)

## Abstract

**Introduction:**

Consumption of unhealthy substances such as tobacco use, and engagement in unsafe sexual activity are the major contributors to morbidity and mortality for pregnant women and the fetus. Although, risky behavior among pregnant women was complex and multifactorial public health concern, there is a limited study on those combined risk behaviors and their predictors among pregnant women in Eastern African countries using machine learning. Therefore, this study was conducted to examine the predictors of risky behaviors among pregnant women in Eastern African countries by using machine learning approach.

**Method:**

The study used a cross-sectional study design from Demograpic and Health Survey data collected from 2012 - 2022 in Eastern African countries. STATA 17 was used for data extraction and preparation. Anaconda 3 and R softwares were used for data preprocessing, and analysis. Model performance was evaluated using accuracy, sensitivity, precision, F1-score and Area Under the Curve (AUC). Finally, the Shapley Additive Explanation (SHAP) was applied to further explain the predictors of risky behaviors among pregnant women.

**Results:**

The pooled prevalence of risky behavior among pregnant women in Eastern African countries were 16.38% (95% CI: 12.63, 20.82). The Light Gradient Boosting Machine (LGBM) achieved an accuracy of 90% and AUC score of 0.96. The analysis revealed that pregnant women who lived in rural areas, being from poor and middle wealth income, women whose husbands had primary education, and women who do not exposed to media were among the most important predictors of risky behavior. Whereas women who are employed, women who utilized more than three Ante natal care service, and women aged between 25 and36 were contributed to a lower predicted likelihood of risky behaviors.

**Conclusion:**

Risky behavior was a complex multifactorial public health challenge in Eastern African countries. The Light Gradient Boosting Machine (LGBM) was the best-performing model for predicting risky behaviors among pregnant women in Eastern African countries. The identified predictors provide valuable insights for developing targeted public health interventions to enhancing and increasing women’s access to more than three Ante natal care visits, enhancing husbands’ educational attainment, extending and implementing targeted media use, and empowering women economically. Expanding media campaigns and community outreach program to promote early and progressive adequate Ante natal care, particularly in rural areas.

**Supplementary Information:**

The online version contains supplementary material available at https://doi.org/10.1186/s12884-026-09725-7.

## Introduction

Health-related risky behavior encompasses actions or personal traits that deteriorate health and well-being both in the short and long term. Behaviors such as smoking and using tobbaco products, engaging in harmful sexual activity, physical inactivity, and consumption of harmful substances are destructive behaviors that leads to poor health outcomes [1, 2]. Currently, the burden of disease shifting from communicable disease to Non-Communicable Diseases (NCDs), which was largely driven by behavioral risk factors [3, 4]. Risky behaviors have a great impact on women’s mental, social, and emotional well-being, leads to premature death arise from cardiovascular diseases, diabetes, cancer, high blood pressure, and respiratory infections [5–7], engaging in one of these behaviors can contribute greatly to developing NCDs [8]. Additionally, risky behavior can increase the risk of chronic diseases, disability, and impair physical and mental condition [9–11]. Further, smoking during pregnancy affects the mother and the fetus, can results in low birth weight, sudden infant death syndrome (SIDS), reduced blood flow, and oxygen level in the baby’s circulatory system. Ultimately, it can results in an increased the risk of miscarriages and premature rupture of membranes [12].

Globally, The prevalence of alcohol use in a combined way was 80% [13, 14], About 185 million drug users, 1.3 billion smokers, and 2 billion alcohol drinkers; of those, 9.8% pregnant women were consumed alcohol. In 2021, approximately. 5.7% of pregnant women use tobacco during pregnancy, and 7.2% were smoking after postpartum period [16], 5.4% use both tobacco and alcohol [17]. Similarly, physical inactivity during pregnancy affects 28% of women [18]. Collectively, 7.2% of all deaths and 7.6% of cardiovascular deaths are linked to physical inactivity [19]. A study showed that substance use in Sub-Saharan Africa accounts for 41.6% and in Central Africa, 55.5%. of those, most commonly used substances are caffeine products 41.2%, tobacco 23.5%, in Brundy 9.9%, Madagascar 19.6% of women were used tobacco products [20]. behaviors such as smoking, excessive substance consumption, and physical inactivity contribute significantly to the burden of disease and morbidity [21, 22].

In order to decrease the burdens of risky behaviors different interventions were conducted for instance the WHO and CDC were conduct a Global School Health Survey (GSHS) to identify behavioral risks and focus on promoting physical activity, healthy diets, and regulating unhealthy food marketing to children [23]. Moreover, many interventions are conducted to get rid of risky behaviors. However, they targeted only a single behavior, while few have addressed multiple behaviors to reduce risky behaviors, the effect is still one of public health burdens [24–30]. Therefore, it is crucial to develop appropriate interventions targeted people to refrain from engaging in risky behaviors. Existing efforts often focus on single issues, such as smoking or unhealthy diets, rather than tackling interrelated behaviors like physical inactivity, substance use, and poor nutrition simultaneously [28, 31, 32].

After a review of previous literature, no studies have been conducted so far by using machine learning models to predict risky behavior among pregnant women in Eastern African countries. Similarly, most of the existing literature focus on specific geographical coverage, and inability to assess the combined effects of multiple risk behaviors in a combined way since, these behaviors shared common and interrelated pathways among one another[33]. Moreover, because of this behaviors are complex and multifactorial, traditional statistical models like logistic regression and linear regression usually fall short in capturing nonlinear patterns and interactions [34–36]. On the other hand, Machine learning can find pattern in data and useful explanations to models through advanced tools like SHAP [37]. Thus, our study used a machine learning approach to predict risky behavior among pregnant women in a combined way in Eastern African Countries, 2012–2022.

## Method 

### Data source and study design

This study employed a cross-sectional design from the DHS dataset. The included countries were, Comoros (2012), Burundi (2017), Ethiopia (2016), Rwanda (2019), Uganda (2016), Zambia (2018), Tanzania (2022), Mozambique (2022), Madagascar (2021), Zimbabwe (2015), Kenya (2022), and Malawi (2016) (Supplementary Fig. 1).

### Source and study population

All reproductive women aged 15-49 years living in the 12 Eastern African countries were the source population. Whereas, all pregnant women living in the 12 Eastern African countries and present in the household during the enumeration period were the study population.

### Inclusion and exclusion criteria

All pregnant women who had provide a valid responses for at least one of the risky behavior indicators during the survey were included in the study. Whereas, pregnant women who were not provide at least one of the risky behavior indicators during the survey were excluded from the study.

### Sampling procedure and sample selection

DHS used a two-stage stratified sampling technique to select representative study participants. The Enumeration Areas (EAs) were chosen using a probability method that was aligned with the size of each area, making sure the selection was done independently in every sampling group. In the next phase, homes were selected in a systematic way. The key demographic and health indicators were collected in each DHS [38]. All the detailed information for the survey is available in Demographic and Health Survey reports from the Measure DHS program website https://www.dhsprogram.com. In the study a weighted total of 199,083 reproductive women were included; of those, 184,746 women were not pregnant during the enumeration period. Finally, this study utilized a weighted sample of 14,337 pregnant women for the final analysis presented the detailed flow chart for the selection of study participants were presented in (Supplementary Fig. 2).

### Study variables

#### Outcome variable

The outcome variable for the study was Risky behavior.

#### Independent variables

The Independent variable include: women’s age, women’s educational level, marital status, women’s occupation, media exposure, husband’s education, wealth status, fuel type, family size, sex of household head, place of residence, and ANC visit.

### Operational definition

*Risky behavior*: was dichotomized into a binary category. Women were considered to be engaging in risky behavior if they exhibited at least one of the following behaviors: using tobacco products, including smoke cigarettes, smoke pipes full of tobacco, snuff by nose, snuff by mouth, smoke kreteks, smoke cigars or cheroots, smoke water pipes, chew tobacco, chew betel liquid with tobacco, and smoke other local substances, and engage in unsafe sexual activity, including not using condom during last sex with partners, having more than one sexual partners excluding spouse, and have been forced into unwanted sexual activity by partner. Whereas, the women were considered not engaged in risk behaviors if they exhibited none of the above behaviors [39, 40].

*Media exposure*: was classified into two categories by merging three variables together. Having mothers listening to the radio, watching TV, or reading magazine. If mothers were exposed at least one of these media sources, were classified as media exposed; otherwise, not media exposed [41].

*Unsafe sexual activity*: was dichotomized by merging three variables together, if the woman were not used condom during the last sex with a partner, having more than one sexual partners excluding spouse, and whether she has been forced into unwanted sexual activity. Whereas, the women were considered not engaged in risky sexual activities if they exhibited none of the above behaviors. Each item was scored as never engaged in the behavior or ever engaged in the behavior in the 12 months before the survey, according to DHS guidelines [15, 42].

### Data management and analysis

Before doing the analysis, weight adjustments were applied to handle the complexity of sampling design and to ensure representativeness using Stata 17. We adjusted the data for both the outcome and predictors using DHS sample weights in all descriptive analyses to ensure a national representativeness of samples. Additionally, anaconda3 and R softwares were used for data preprocessing, missing values management, and for conducting the analysis. This study used a machine learning based approach frameworks. The steps employed include data preprocessing, model selection, model building, model evaluation, parameter tuning, and making predictions [43].

### Data preprocessing

**Fig. 1:**
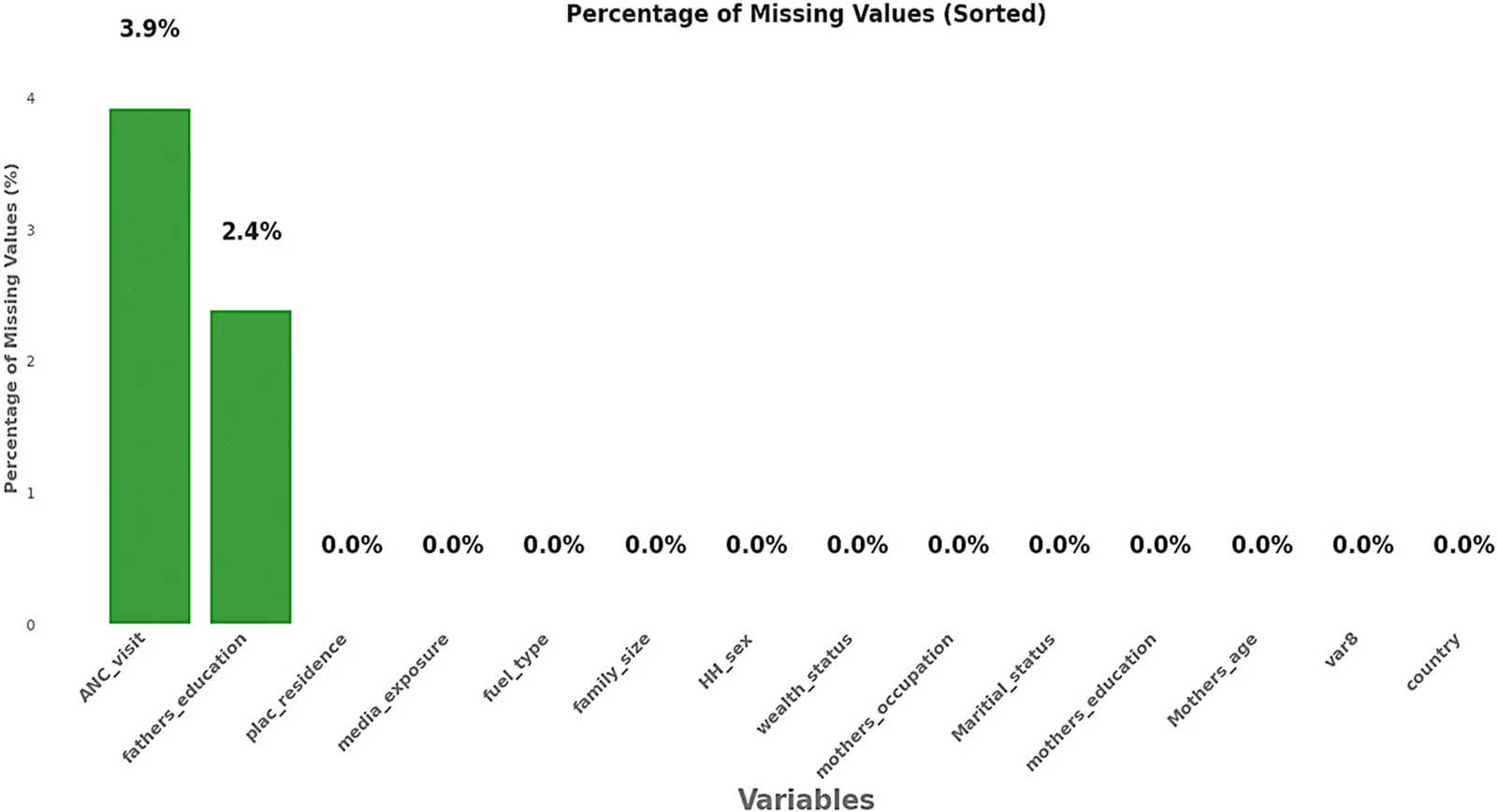
Percentage of missing variables before K-Nearest Neighbor imputation

Data were preprocessed by encoding the data to numeric for computer manipulation. Missing values in the dataset were imputed by using the K-nearest neighbors (KNN) imputation technique. Since, the KNN approach worked by the concept of distance-based techniques to impute missing data, the KNN is effective for both categorical and continuous variables, and it easily handle many missing values effectively [44]. Percentages of missing value include father’s education (2.4%) and ANC visits (3.9%) (Fig. 1**)**. Moreover, multicollinearity was evaluated by using the correlation coefficient matrix; high multicollinearity was defined as having a correlation coefficient more than 0.8.

### Dimensionality reduction

The Boruta algorithm was employed in this study as a dimensionality reduction technique to enhance model performance and reduce data complexity. Boruta iteratively confirms or disproves attributes, ensuring that only significant predictors are retained. Non-important variables were rejected by the algorithm, and increased model efficiency, and improve performance [45].

### Feature engineering

Feature engineering including the process of categorizing, and encoding the most relevant characteristics from the available datasets to build machine learning models [46]. This study employed one-hot encoding for nominal variables and label encoding for ordinal variables [47]. Both methods of feature engineering were implemented in this study.

### Data balancing

The SMOTEENN method was used to address class imbalance in the dependent variables. It first generates additional synthetic samples for the minority class by including the existing samples within the feature space which helps to reduce overfitting. Because SMOTEENN is robustly better than SMOTE in terms of accuracy and mean squared error across the same sample, improved generalization. Moreover, cross-validation analysis reveals that SMOTEENN achieves lower standard deviation compared to SMOTE, indicating more stable and reliable performance. Hence, SMOTEENN is a more effective method for handling class imbalance [48]. Since the unbalanced dataset distorted our results as a result, we used the balanced dataset to train the model [49].

### Data split

To prevent the effect of model overfitting. We employed a 10-fold cross validation technique to train and test the data. Since 10-fold validation provide reliable results since most of the data 80% was used for train the model. The model learned from the training data (80%). While 1-fold (20%) was used to test the model. In order to train the prediction function, k-fold splits all observations into equal-sized sample groups called folds and k-1 folds. The remaining fold is then used for testing k times in a row. The average of the values calculated in the loop is the k-fold cross-validation performance metric.

### Model selection

An appropriate classifier was selected based on the previous literatures that was conducted on classification and prediction. The selection of these algorithms was based on their suitability for classification tasks and their compatibility with the characteristics of our dataset. Include RF, LR, LGBM, Naive Bayes, ANN, DT, XGB, KNN, AdaBoost, SVM, CatBoost [50–52].

### Model building and evaluation

The models were trained on 80% of the data and the rest were used for testing (20%). Sensitivity, specificity, F1-score, precision, accuracy and AUC, were used to evaluate the performance of the models. For this study accuracy and AUC were implemented to measure and evaluate models with balanced dataset [53]. Accuracy measures the overall correctness of the model’s predictions. It is the ratio of the number of correct predictions to total predictions. Precision quantifies the accuracy of positive predictions made by the model. It finds the ratio of true positive predictions to total predicted positives. Recall, also known as sensitivity or true positive rate, assesses the model’s ability to identify all positive instances; it is effectively utilized in various computational models and machine learning applications [54]. Principally we used the AUC score to measure the performance of predictive models since AUC is an alternative measure other than accuracy to evaluate the performance of models, since it is threshold-independent and has the ability to provide a more overall performance for predictive models (Table 1) [55].

**Table 1:** A two by two tables for the predictions of risky behaviours among pregnant women

Classes	Predicted class		
	Yes	No	Total
Yes	TP	FN	TP + FN
No	FP	TN	FP + TN
Total	TP + FP	FN + TN	TP + FN+FP + TN

As illustrated in Table 1 two by two tables, the following formula were calculated.

1. Accuracy = TP + TN /TP + TN + FP + FN.
2. Sensitivity (Recall) = TP /TP + FN.
3. Specificity = TN /TN + FP.
4. Precision = TP/ TP + FP.
5. F1-score = 2TP/2TP+FP+FN.

The above metrics used to comprehensively evaluate the performance of each predictive model in terms of overall correctness, accurate positive predictions, and identification of positive instances, balanced measure, and discriminatory ability.

### Parameter tuning

Hyperparameter is an external adjustment to the model parameter for best combination of performance whose value were set by the user [56]. The model was optimized with the best parameters by applying the Optuna with 10-fold cross validation on the stratified search space with one hundred trials.

### Model explanation 

**Fig. 2:**
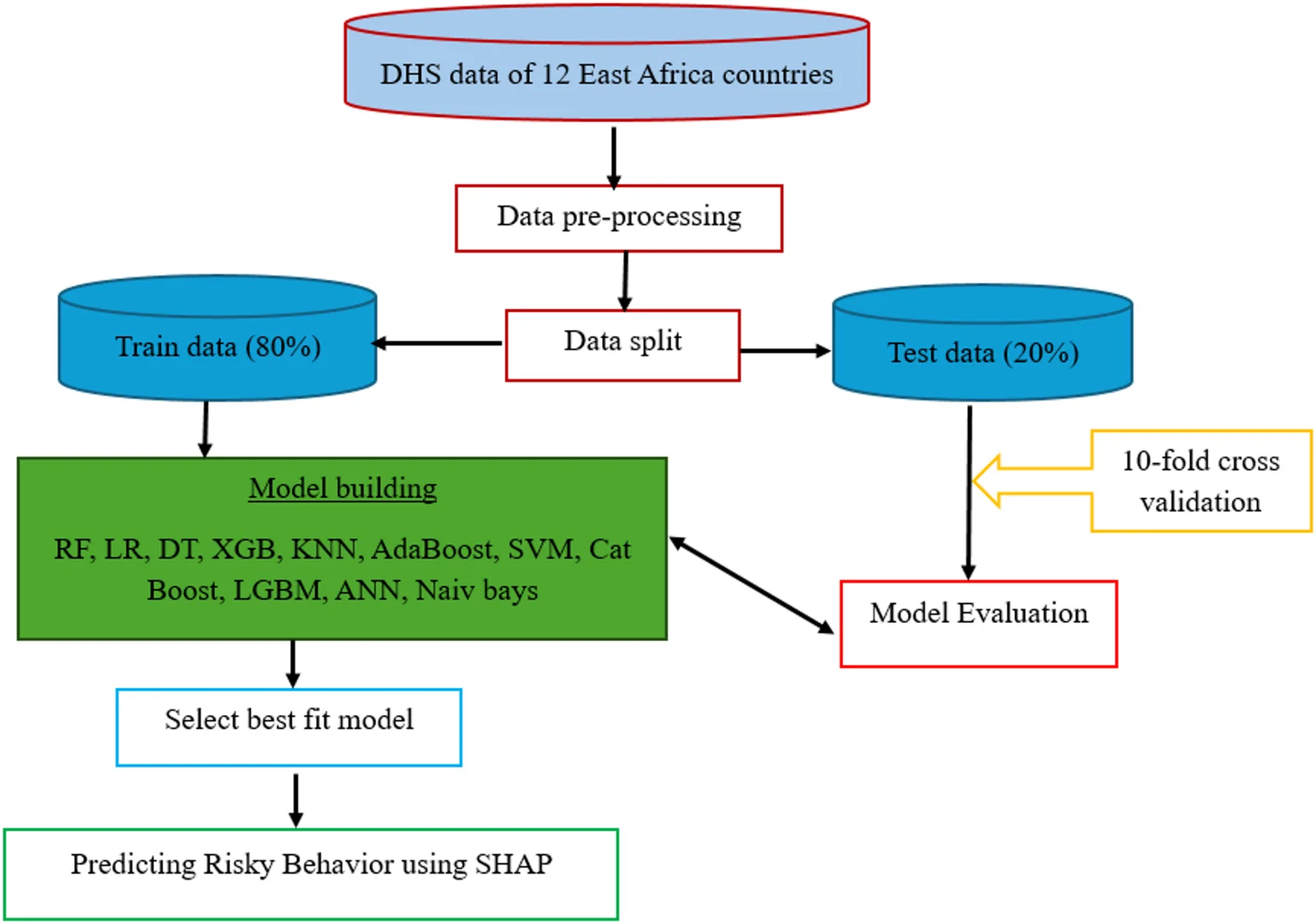
Workflow diagram taken to conduct the study

The interpretability of machine learning models were essential to ensure clinical relevance and support evidence-based decision-making. To address the complexity of black-box model of advanced models. SHAP analysis offers a unified and theoretically grounded method to explain individual predictions by fairly attribute the contribution of each feature to the model’s output [57]. SHAP played a central role in both feature selection and model interpretation. Features were levelled by considering their average absolute SHAP values, which handle to identify and the variability of the outcome variables. Moreover, a range of SHAP visualization techniques were used to improve both global and local model interpretability. The global feature importance plot provided an overall ranking of feature. Similarly, the Beeswarm plot illustrated the direction and distribution of features across the population, and the waterfall plot offered detailed case-level explanations. These tools collectively facilitated the discovery of complex interactions and non-linear relationships between the predictors that are often overlooked using traditional statistical methods. In this study, the integration of SHAP provided a comprehensive analytical framework for understanding health risk behaviors. SHAP enabled transparent and data-driven explanations of model prediction, the workflow was illustrated in (Fig. 2).

## Result

### Socio-demographic and behavioral related characteristics 

Majority of the respondents have attained primary education 47.7%, and 20.7% of the participants were with no education. About 36.7% were categorized as rich, and 44.5% of the participants were poor. The majority of participants were resides in rural areas 74.8%. More than half of the respondents 54.5% were currently working, 16.56% of the participant were engaged in some form of risky behaviors, from those approximately 14.75% of participants were involved in unsafe sexual activity, while 2.16% of participants were using tobacco products. All the detailed descriptions of study participants are presented in Table 2. 

**Table 2:** Socio-demographic and Behavioral related characteristics of study participants in Eastern African countries, 2012–2022 (*n* = 14,337)

Variables	Categories	Frequency (weighted)	Percentage	
Women’s age	15–24	6197	43.22	
	25–36	6839	42.70	
	≥37	1301	9.08	
	Total	14,337	100	
women’s education	No education	2957	20.62	
	Primary	6846	47.75	
	Secondary & Higher	4534	31.62	
	Total	14,337	100	
Marital Status	Single	1061	7.40	
	Married	12,564	87.63	
	Divorced/separated	712	4.97	
	Total	14,337	100	
Women’s Occupation	Not-working	6522	45.49	
	Working	7815	54.51	
	Total	14,337	100	
Husband’s Education	No-Education	2981	20.79	
	Primary	6446	44.96	
	Secondary & Higher	4910	34.25	
	Total	14,337	100	
ANC Visits	< 3	13,678	95.4	
	≥3	659	4.6	
	Total	14,337	100	
Wealth status	Poor	6383	44.52	
	Middle	2688	18.75	
	Rich	5266	36.73	
	Total	14,337	100	
Household head sex	Male	11,313	78.91	
	Female	3024	21.09	
	Total	14,337	100	
Family size	1-4	7022	48.98	
	5– 8	5899	41.15	
	≥9	1416	9.88	
	Total	14,337	100	
Fuel type	Non-Electricity	13,635	95.10	
	Electricity	702	4.90	
	Total	14,337	100	
Media exposure	No	5427	37.85	
	Yes	8910	62.15	
	Total	14,337	100	
Place of residence	Rural	10,736	74.88	
	Urban	3601	25.12	
	Total	14,337	100	
Risky behavior	Yes	2374	16.56	
	No	11,963	83.44	
Unsafe sexual activity	Yes	2115	14.75	
	No	12,222	85.25	
Tobacco use	Yes	309	2.16	
	No	14,028	97.84	
Country	Burundi	1348	9.40	
	Ethiopia	1122	7.83	
	Kenya	1864	13	
	Comoros	340	2.37	
	Madagascar	1285	8.96	
	Malawi	1833	12.79	
	Mozambique	945	6.59	
	Rwanda	843	5.88	
	Tanzania	1165	8.13	
	Uganda	1872	13.02	
	Zambia	1109	7.74	
	Zimbabwe	611	4.26	

### Prevalence of risky behavior

The pooled prevalence of risky behavior among pregnant women in Eastern African countries was 16.38% (95% CI: 12.63, 20.82). Madagascar had the highest prevalence of risky behavior (26.46%, 95% CI: 24.05%–28.87%), and Ethiopia had the lowest prevalence (4.46%, 95% CI: 3.25%–5.66%). The detailed prevalence for each country is provided using a Forest Tree plot in (Supplementary Fig. 3).

### Sensitivity analysis

Based on the country-level sensitivity analysis result, the effect sizes for each country are consistently strong, and heterogeneity between the countries provides a reliable estimate, suggesting that the observed positive effect is a consistent and generalizable finding across the included nations, and the pooled estimated prevalence of risky behavior is between 12.63% and 20.86% after the deletion of a single country, as illustrated in (Supplementary Fig. 4). Similarly, a sensitivity analysis by DHS year was examined using the random-effects model to assess transportability. Based on the findings, no countries had a significant influence on the pooled estimated effect size. When the countries were omitted, the pooled estimated prevalence fell within the 95% CI of the total effect size (Supplementary Fig. 5).

### Machine learning analysis results

#### Feature selection

**Fig. 3:**
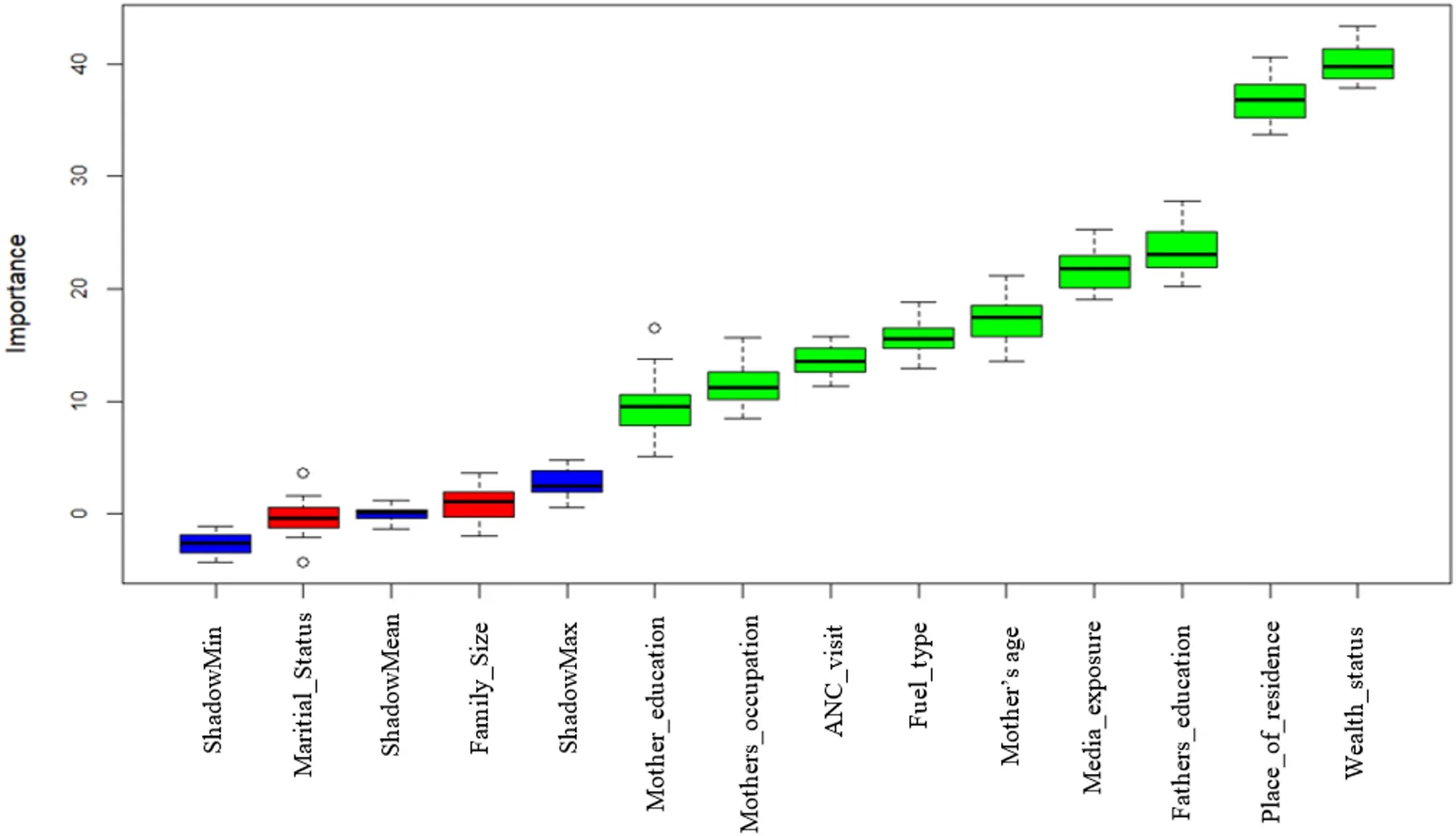
Boruta feature importance plot for the predictors of risky behavior among pregnant women

Boruta analysis identified significant predictors of risky behavior such as, place of residence, mothers age, fuel type, wealth status, husband education, ANC visit, media exposure, mothers' occupation, mother s education were confirmed by the algorithm and included in the next analysis. However, marital status and family size were rejected (Fig. 3).

#### Data balancing

**Fig. 4:**
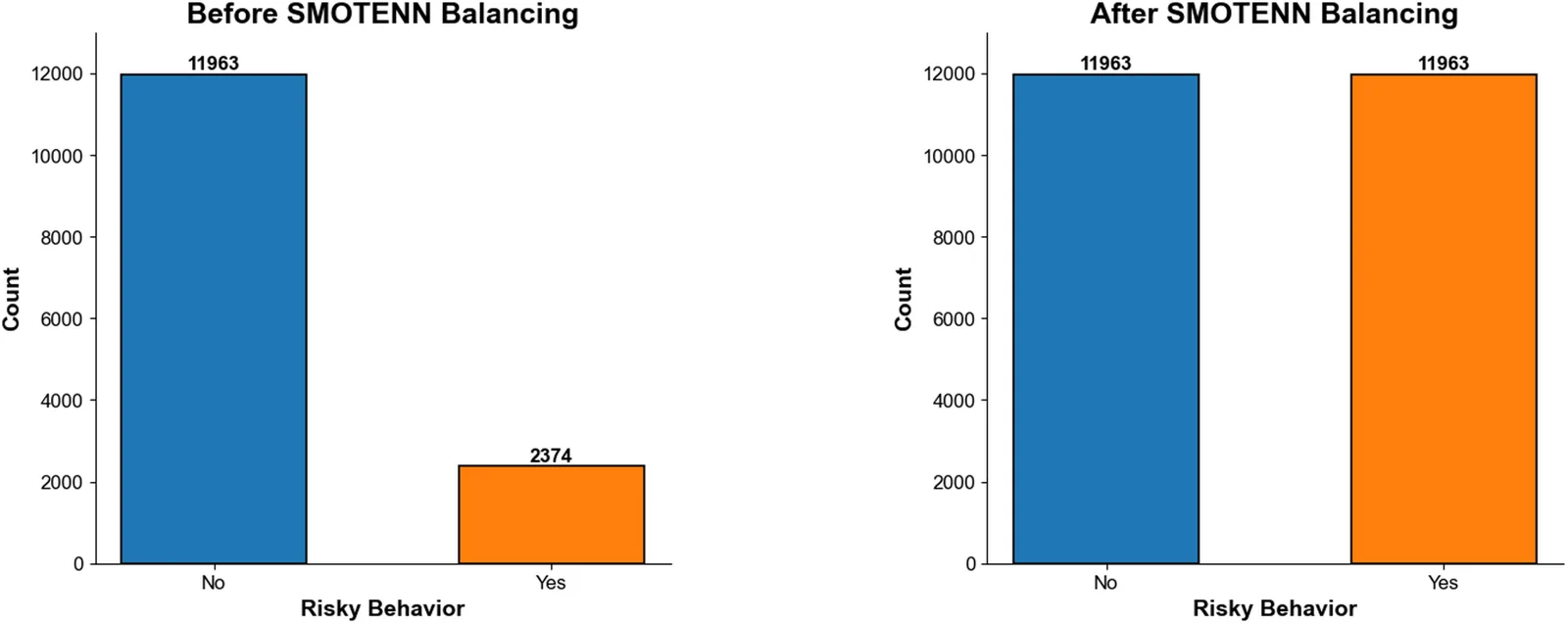
Distribution of risky behavior among pregnant women before and after data balancing using SMOTENN balancing technique

The SMOTENN algorithm was used to solve the problem of class imbalance in the outcome variable. Before balancing, the majority of the data, 11,963, may determine the minority data, 2374; hence, to avoid the domination of the majority data and to balance the unbalanced class, the SMOTEENN oversampling technique generated 9589 additional synthetic data for minority classes in the training set. Hence, the data was changed from unbalanced to balanced distribution for both classes (Fig. 4). The updated proportions greatly decreased the discrepancy while maintaining data integrity. This result was obtained by combining artificial oversampling of the minority class with ENN’s elimination of noisy or ambiguous values from the majority class. Consequently, the balanced dataset made it easier to train a more accurate model (Fig. 4) [48].

### Model building and evaluation 

Based on the comprehensive evaluation of model performance metrics, LGBM model demonstrates the highest performance among other models, achieving the accuracy of 90% and AUC score of 0.96 (Table 3, Supplementary Fig. 6).

**Table 3:** Evaluation metrics of balanced dataset for predicting risky behavior among pregnant women in Eastern African countries, 2012–2022

No	Model	Accuracy (%)	F1 score	Precision	Sensitivity	Specificity	AUC
1.	SVM	89	0.88	0.93	0.84	0.94	0.95
2.	LGBM	90	0.89	0.94	0.87	0.92	0.96*
3.	AdaBoost	83	0.81	0.92	0.73	0.94	0.91
4.	CatBoost	88.9	0.90	0.90	0.84	0.92	0.95
5.	XGB	89.5	0.83	0.89	0.85	0.93	0.95
6.	ANN	92	0.91	0.94	0.89	0.74	0.95
7.	KNN	84	0.84	0.60	0.84	0.83	0.93
8.	RF	90	0.90	0.94	0.87	0.91	0.90
9.	DT	87	0.87	0.87	0.87	0.87	0.90
10.	NB	78	0.65	0.84	0.69	0.71	0.85
11.	LR	82	0.88	0.93	0.83	0.94	0.94

Hint: Maximum performance - *, *ANN * Artificial neural Networks, *AUC * Area under the Curve, *DT * Decision Tree, *LGBM* Light Gradient Boosting Machine, *KNN * K-nearest neighbor, *LR * Logistic Regression, *RF * Random Forest, *SVM * Support Vector Machine, *XGB * Extreme Gradient Boosting, *NB * Naive Bayes

**Fig. 5:**
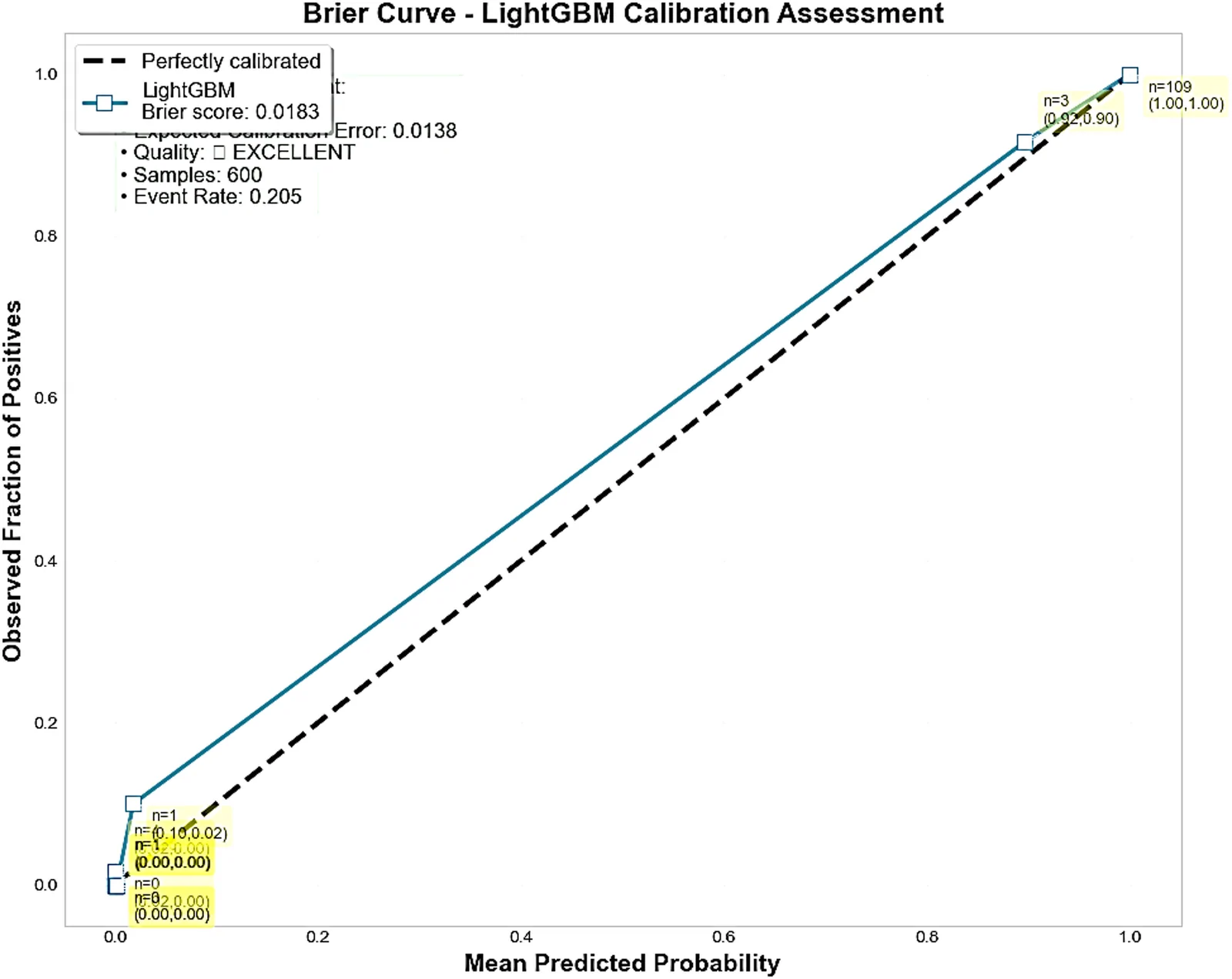
Brier curve for LGBM model for predicting risky behavior among pregnant women in Eastern African countries

The LGBM model achieved better performance in predicting probabilities. With a Brier score of 0.0183, the model achieved excellent calibration, make its predictions more accurate and reliable. The model predicted an average probability of 19.2% for positive cases, which closely matches the actual rate of 20.5% observed in the test data of 600 samples. This close alignment between predictions and reality across all probability levels. These strong results validate the model’s readiness for practical applications and research use. The excellent calibration performance means that when the model predicts a 30% chance of an event occurring, we can be confident that approximately 30% of such cases will actually occur (Fig. 5).

### Parameter tuning

The LGBM model was the best-performing model in terms of accuracy and AUC score. To further optimize we applied the Optuna framework as provided in Table 4.

**Table 4:** Default and optimal parameters for LGBM model

No	Parameters	Default value	Optimal value
1	boosting_type	‘gbdt’	‘dart’
2	objective	regression	binary
3	metric	12	auc
4	n_estimator	100	300
5	num_leaves	31	100
6	learning_rate	0.1	0.3
7	max_depth	-1	7

### Predicting risky behaviors 

After optimizing, the model were tested on 2391 sample test data. From 2391 pregnant women who were engaged in risky behavior, the model predicted 2175 women correctly as engaged in risky behavior (true positive), and from 2391 pregnant women who were not engaged in risky behavior, the model predicted 2247 women as not engaged in risky behaviors (true negative). However, the model misclassified 144 non-risky women as risky (false positive) and 216 risky women as non-risky (false negative) (Table 5).

**Table 5:** Two by two table of optimized model for the predictions of risky behavior among pregnant women in Eastern African countries

Classes	Predicted class	
	Positive prediction	Negative prediction
Positive class	True positive (TP) 2175	False Negative (FN) 144
Negative class	False Positive (FP) 216	True Negative (TN) 2247

### SHAP analysis result

**Fig. 6:**
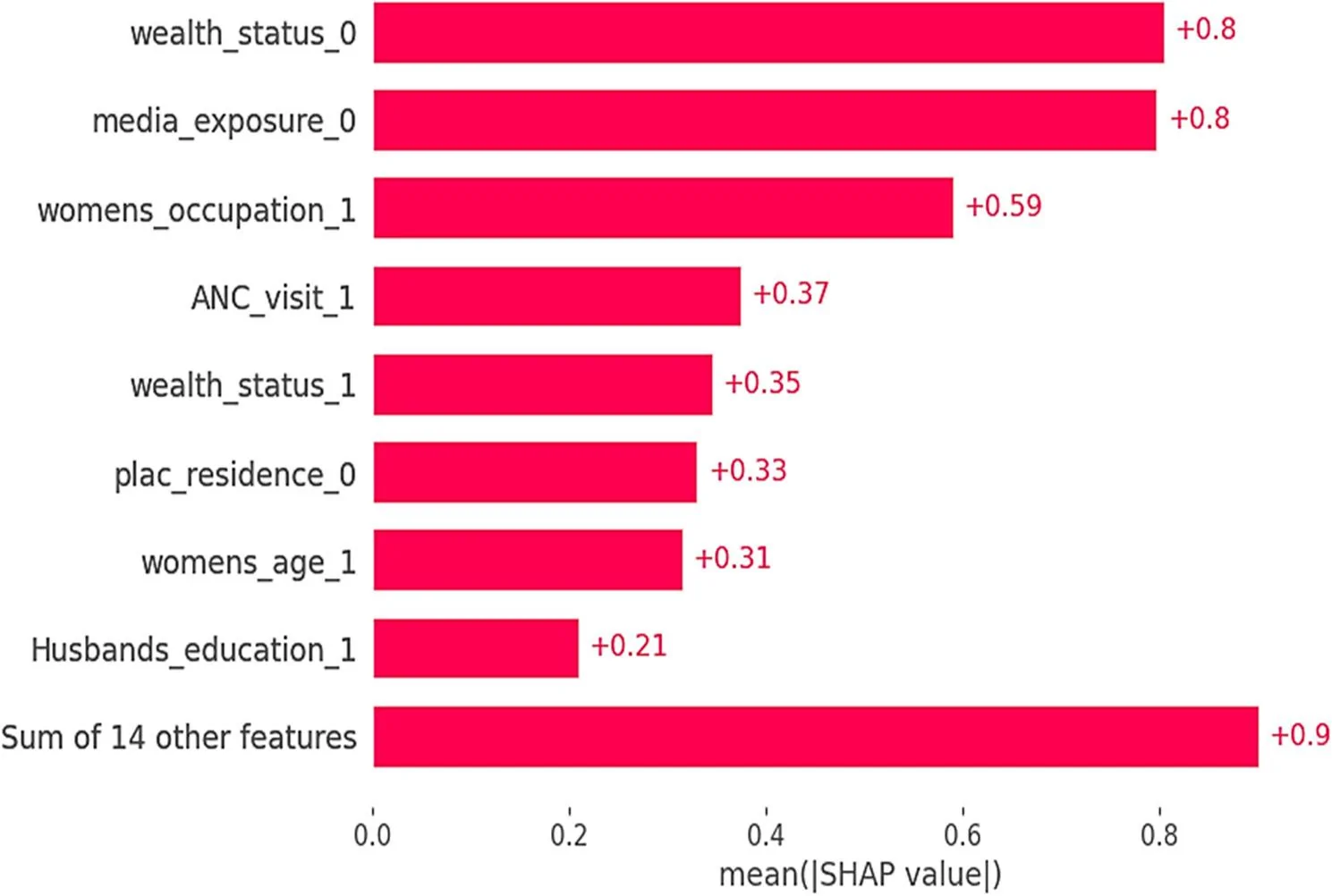
SHAP global importance plot for the predictions of risky behavior among pregnant women in Eastern African countries (**Hint**: *Wealth status_0 = poor wealth income, media_exposure_0 = exposed to media, ANC visit_1 = utilize > = 3 ANC services, wealth status_1 = middle wealth income, place residence_0 = rural, women’s occupation _1 = employed, husbands education_1 = primary, women’s age = 25–36*)

The mean absolute SHAP value is shown on the x-axis, and the associated features are listed on the y-axis based on the result variable with more mean absolute SHAP values or variables appear on top of the bar reveal the most important predictors: women who lived in rural area, women’s with poor wealth income, women’s with middle wealth income, husbands education being primary, women’s who are employed, women’s who are not exposed to media, women’s who utilize more than three ANC services( > = 3), women’s whose age between 25–36 were the top most important variables sorted in descending order from higher to lower (Fig. 6).

### SHAP beeswarm plot

**Fig. 7:**
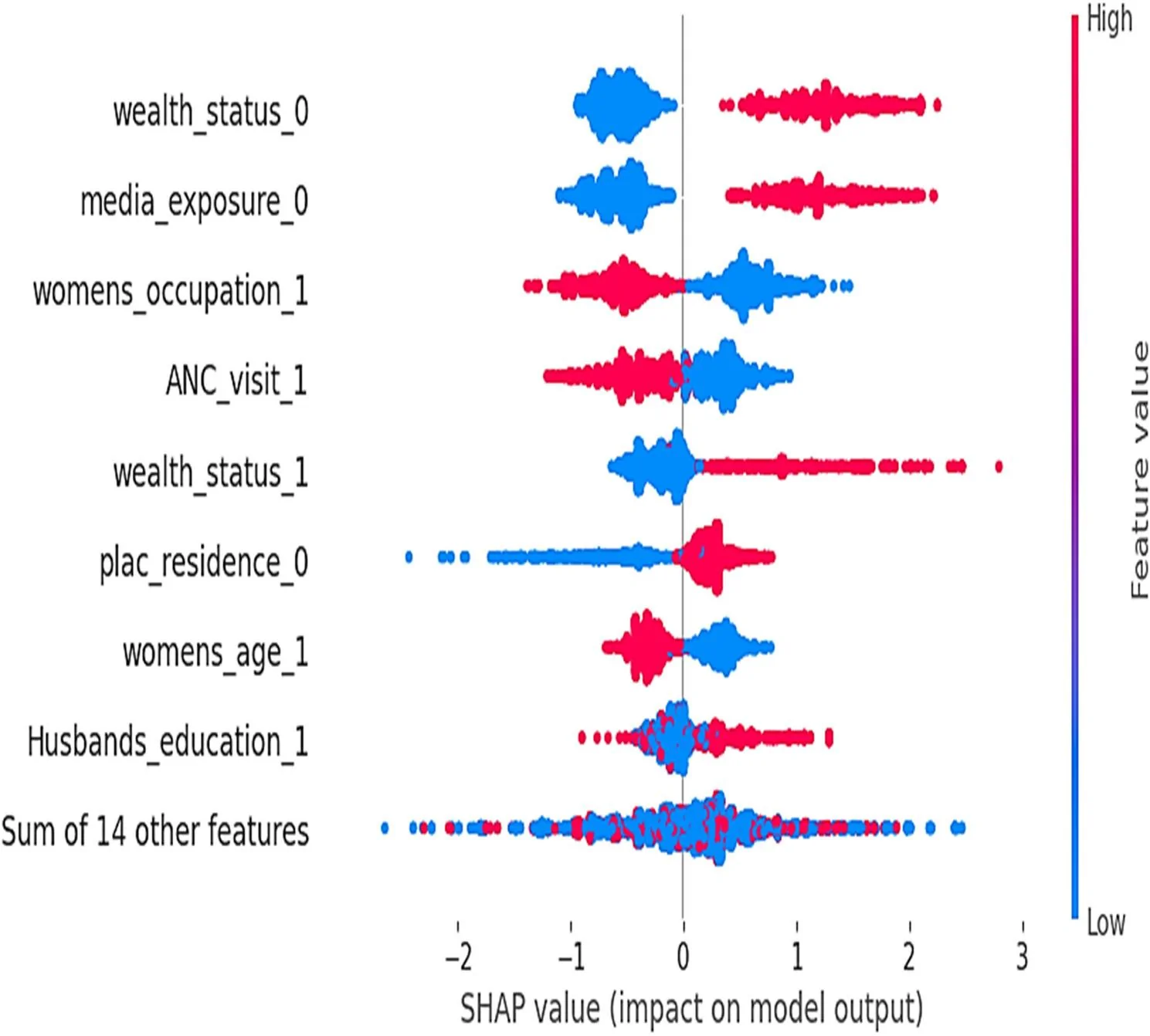
The beeswarm plot for the prediction of risky behavior among pregnant women in Eastern African countries (**Hint**: *Wealth status_0 = poor wealth income, media_exposure_0 = exposed to media, ANC visit_1 = utilize > = 3 ANC services, wealth status_1 = middle wealth income, place residence_0 = rural, women’s occupation _1 = employed, husbands education_1 = primary, women’s age = 25–36*)

The analysis was identified women who lived in rural area, women’s with poor wealth income, women’s with middle wealth income, husband’s education being primary, and women’s who are not exposed to media increase the likelihood of predicting risky behavior among pregnant women. Whereas women’s who are employed, women’s who utilized more than three ANC services, and women’s whose aged between 25 and 36 lowers the likelihood of predicting risky behaviors among pregnant women (Fig. 7).

## Discussion

The study aimed to predict risky behaviors among pregnant women in Eastern African countries using various machine learning models applied on the DHS data. The LGBM model were performed better than other models. Hence, The LGBM was the best predictive model and further analysis was performed after LGBM demonstrated a strong ability to distinguish between women with and without risky behaviors. Similarly, a study conducted in China suggested that gradient boosting is the best model to predict risky sexual behaviors among university students. This consistency supports the robustness and generalizability of the LightGBM algorithm across different populations and behavioral outcomes [58].

Based on the study finding that women living in rural areas had a higher predicted probability of engaging in risky behaviors compared to urban counters. This finding is consistent with a study conducted in Virginia [59], Myanmar [60] and Ethiopia [39]. This might be explained by, the traditional lifestyles and poor quality of life in rural areas, limited access to health education, insufficient prenatal care services in rural areas, cultural norms that may promote risky behavior, and lack of public health initiatives in rural areas could be contributing to increase women's engagement in risky behavior [61, 62].

Additionally, womens from lower and middle wealth indices were associated with higher predict probablity to engaging in risky behaviors. There is a similar finding with study conducted in Canada [63], Denmark [64], and Pomerania [65]. This could be due to the fact that financial constraints may limit access to health-related information,poor access to wholesome food, and Essential health care services like, pre/post pregnancy related care services which then leads to stress and frustration due to financial hardship which may contribute the womens to engaged in to some form of risky behaviours [66].

Moreover, paternal education being primary increases the predicted probability of risky behaviors among pregnant women. This finding aligns with other studies conducted in Uganda [67] and Australia [68], showing that paternal education level significantly affects maternal health conditions. This might be explained by lower educational attainments among husbands might be associated with the lower recognition and managment of early pregnancy signs and symptoms, lower health care decision making ability, decreased support and counseling for pregnancy complications and risks, and insufficient support for maternal healthful behavior [39, 69].

Furthermore, women who were not exposed to the media were associated with a higher predicted likelyhood of engaging in risky behavior [70]. This may be due to media is an important medium to transmitting essential public health messages, such as adequate dietary diversity intake for pregnant mothers as well as for the fetus, promoting ANC service utilization, promoting environmental hygiene and sanitation practices, raising awareness among women on early pregnancy complications, and strengthening ANC care practices [71]. 

In contrast, women who are employed or working were associtated to decrease the predicted likelihood of engaging in risky behaviors. This finding was similar with a study conducted in Australia [68] and Iran [72]. This might be explained by occupation can strengthen women’s finances and increase their reliance on making a healthy lifestyle, reducing stress arise from finincial hardship, including utilization of ANC care services on a timely basis, and promoting better healthful behaviors [68].

Similarly, women who attend more than three ANC care services were lower predicted probablity to engage in risky behaviors. This finding is aligned with other studies conducted in Iran [73] and Pomerania [65]. This could be due to the fact that ANC visits during pregnancy should encouraging and providing opportunities for health education, early detection of pregnancy complications, empowering women’s to have adequate knowledge about proper nutrition and good behaviours, substance use and provide counseling service on nutrition and substance avoidance, and empowering women’s to make better health decision all of which contribute to reducing risky behaviors [74, 75].

Finally, older women aged between 25 and 36 were associated with lower predicted probablity to engage in risky behaviors. This finding is supported by studies conducted in Denmark [64], Ethiopia [76], Scotland [6], and South Africa [28, 77]. This could be explained as younger women (< 25 years) may lack adequate life experience to engaged in to risky behaviors, while older women may have lower probability of engaging in unsafe sex, transactional sex, use of harmful substance, and peer pressure influence, this age group is strongly associated to higher maturity, self-regulation since they have strong background for unhealthy patterns, better ability for impulse control and unwanted behaviors than younger women [78, 79].

### Strength and limitations

This study employed a standardized measurement to define tobacco use and unsafe sexual behaviors, which are internationally recognized and adopted by the WHO as a standardized tool for measuring this behaviors in this study. Consequently, comparison of results across many studies will be easy. Similarly, the study used SHAP model interpretation techniques, which enable us to interpret complex models accurately, which is based on an optimized and best-performing model, which increases model performance to accurately predict risky behaviors among pregnant women.

In conrast, the study design was cross-sectional; it does not show a causal relationship between the variables (cause-and-effect). Similarly, it also does not show long-term effects. In addition, our study was only limited to tobacco use and unsafe sexual activity to measure risky behaviors among pregnant women since other variables like alcohol use and physical activity were not available and collected in the DHS program. We recommend other researchers to include them in the future, and include subgroup analysis to explore behavior-specific determinants. Moreover, the secondary nature of the dataset makes it difficult to explore and find additional essential features to accurately predict risky behavior comprehensively.

## Conclusion

The LGBM achived better predictive performance to predicting risky behavior among pregnant women. Similarly, the findings revealed that women who lived in rural areas, women with poor wealth income, women with middle wealth income, husbands’ education being primary, and women who do not exposed to media were associated with a higher predicted likelihood of risky behavior among pregnant women. Incontrast, women who are employed, women who utilize > = 3 ANC service, and women aged 25-36 were associates with a lower predicted likelihood of risky behaviors among pregnant women. To minimize the burden of risky behaviors interventions should concentrate on enhancing women’s access to more than three ANC service, enhancing husbands’ educational attainment, implementing targeted media use, and empowering women economically. Media campaigns and community outreach program to promote early and progressive ANC care, particularly in rural areas; combining healthcare access and economic empowerment programs for pregnant women, lastly, creating health communications that take educational disparities into consideration.

### Implication of the study

The findings of this study have important contributions for public health practice and policy. In the health sector, the developed predictive model of machine learning can be used for improving maternal and child health to predict risky women’s behavior. Health ministries can use the result to efficiently allocate limited resources and reduce risky behaviors during pregnancy. For the communication and media sector, this finding can be used to develop effective health communication strategies, mainly for areas with limited access to health information. For the technology sector, the study may help to develop user-friendly digital tools and mobile applications to increase access to information for pregnant women and support maternal health monitoring. The economic and social development sectors can use these findings to promote women’s employment and financial empowerment to reduce risky behaviors.

## Supplementary Information

Supplementary Material 1.

## Data Availability

All relevant data are publically available in the DHS program website (https:/www.dhsprogram.com) and additional files are available in supplementary files (Supplementary Material 1).

## Abbreviations

ANC: Ante Natal Care
ANN: Artificial Neural Networks
AUC: Area Under the Curve
CV: Cross Validation
DHS: Demographic and Health Survey
DT: Decision Tree
LGBM: Light Gradient Boosting Machine
KNN: K-Nearest Neighbor
LR: Logistic Regression
ML: Machine Learning
RF: Random Forest
ROC: Receiving Operating Characteristics
SHAP: Shapely Additive Explanation
SVM: Support Vector Machine
SMOTEENN: Synthetic Minority Oversampling Techniques with the Edited Nearest Neighbor
WHO: World Health Organization
XGB: Extreme Gradient Boosting
