# Supplementary material for: Predicting Risky Behavior Among Pregnant Women in Eastern African countries using Pooled Demographic and Health Survey (DHS) Data: An Explainable Machine Learning Approach
Source: BMC Pregnancy Childbirth. 2026 Aug 10;26:1276. doi: 10.1186/s12884-026-09725-7 (PMC13737786; doi:10.1186/s12884-026-09725-7)
Supplement: Supplementary file 1 [file 12884_2026_9725_MOESM1_ESM.docx]

**Supplementary Materials**

**Supplementary Figure 1**. Map of study area Eastern African Countries to predict risky behavior among pregnant women, 2012-2022

**Supplementary Figure 2**. Sample size selection flow chart to identify the determinants of risky behavior among pregnant women, 2012-2022.

**Supplementary Figure 3**. Forest plot depicting the allover prevalence of risky behavior among pregnant women in Eastern African countries, 2012-2022.

**Supplementary Figure 4**. Sensitivity analysis by country for the risky behavior among pregnant women, in Eastern African countries, 2012-2022

**Supplementary Figure 5**. Sensitivity analysis by DHS year for the risky behavior among pregnant women, in Eastern African countries, 2012-2022

**Supplementary Figure 6***.*  ROC curve analysis of selected machine learning algorithms to predicting risky behavior among pregnant women in Eastern African countries, 2012-2022

**Supplementary Figure 7.** DHS Permission Letter
